# High-Precision Chromatic Confocal Technologies: A Review

**DOI:** 10.3390/mi15101224

**Published:** 2024-09-30

**Authors:** Jingwen Li, Rui Ma, Jiao Bai

**Affiliations:** 1 Tsinghua-Berkeley Shenzhen Institute, Tsinghua University, Shenzhen 518055, China; lijw23@mails.tsinghua.edu.cn; 2 Shenzhen International Graduate School, Tsinghua University, Shenzhen 518055, China; marui@sz.tsinghua.edu.cn; 3 Institute of Materials, China Academy of Engineering Physics, Mianyang 621907, China

**Keywords:** chromatic confocal technology, high-precision measurement, displacement measurement, contour measurement, online measurement

## Abstract

Chromatic confocal technology is widely used for precise, steady, and efficient displacement measurement in many industrial fields. It employs the confocal and dispersion principles to encode axial positions with the wavelengths of the reflected broad spectrum. The typical chromatic confocal sensor includes a light source, a dispersion objective, conjugate pinholes, and a spectral detection device. This study offers an overview of the current research on chromatic confocal technology. Because of its good performance in displacement detection, chromatic confocal technology has been widely used in contour measurement, biomedical imaging, and thickness measurements, as part of global and professional research. Due to its structural flexibility, it is also easily integrated into industrial equipment for in-machine and online profile measurements. It holds significant potential for future applications in industrial manufacturing and scientific research. However, there are also some challenges to be explored in terms of the broadband light source, dispersive optics design, and the balance between speed and accuracy in signal processing.

## 1. Introduction

Since entering the 21st century, the manufacturing industry has developed rapidly towards high performance, digitization, and intelligence [1,2,3]. In the manufacturing field, precise measuring technology acts as the fundamental tool for dimension characterization, positioning, defect detection, etc. [4,5,6,7]. It plays a key role in enhancing the comprehension and optimization of manufacturing processes and products [8]. Among the various types of measuring technologies, high-precision displacement sensors are widely used for detecting the displacement of a workpiece or scanning the contour before or after manufacturing [9,10,11]. For precise semiconductor manufacturing in particular, nanometer-level displacement measuring performance is required for workpiece positioning to meet the accuracy and repeatability requirements of fine photo-lithographic patterns [12,13,14,15]. In addition, high-precision measuring technology is also applied to the morphology and thickness of photovoltaic glass, integrated circuit protective layers, and anti-reflective thin films [16,17].

In addition to traditional contact probe measuring methods, non-contact sensors are increasingly being used in the high-precision manufacturing field [18]. Compared with contact methods, non-contact measurement avoids physical contact between the probe and the measured object, which is advantageous in relation to non-destructive, non-deforming, and efficient measuring requirements. Common non-contact sensors utilize electromagnetic, optical, or ultrasonic principles for the displacement measurement [19,20,21]. For example, electromagnetic displacement sensors have high measurement precision and robust structural durability, but they are also susceptible to electromagnetic interference and need a bigger sample surface than the probe’s diameter [22,23,24,25,26]. Laser triangulation sensors project a laser beam onto the sample surface to determine the position from the reflected light based on the geometric relationship of the optical space configuration [27,28,29]. However, the precision is limited and easily disturbed by different measuring materials and surface inclines [30,31]. Laser interferometers and grating interferometers can realize multi-degree-of-freedom measurements [32,33,34,35] with sub-nanometer precision [36,37,38,39]. Laser interferometers detect the displacement from the laser phase shift of the target surface [40,41,42], requiring a highly steady environment [43,44,45,46,47]. Grating interferometers can achieve absolute displacement measurement [48,49,50,51] and angle measurement [52,53,54,55], but they are affected by the grating quality and signal processing ability [56,57,58]. Further, interferometers have relatively complex structures [59,60,61], making signal analysis, miniaturization, and integration difficult [12,62,63,64]. Robust signal resolution algorithms can improve the efficiency and accuracy of displacement measurement [65,66,67]. Usually, the grating is the critical component in grating interference measurements and is undertaken to improve the efficiency and accuracy of displacement measurements. The grating can be manufactured by laser interference lithography, holographic lithography, etc. [68,69,70,71]. The structured light method is also a commonly used measuring method in the vision and 3D reconstruction industry [72,73,74,75,76]. With advanced algorithms, such as machine learning [77,78,79,80,81], we can obtain real-time online measurements with ordinary precision [82,83,84,85,86].

Chromatic confocal sensing technology is also a non-contact optical measuring method with nanometer-level displacement resolution, a wide displacement range, high tolerance for measurement angles, and excellent integrability. It utilizes the principle of chromatic dispersion to focus different wavelengths at different axial positions. By projecting a broadband light onto the sample surface, chromatic confocal sensors can precisely extract the position or layer thickness by analyzing the reflected light spectrum. At present, chromatic confocal sensors can typically provide the measurement results in several microseconds. Hence, they are used increasingly often as a better replacement for laser triangulation sensors, in both displacement and contour measurement applications.

**Figure 1:**
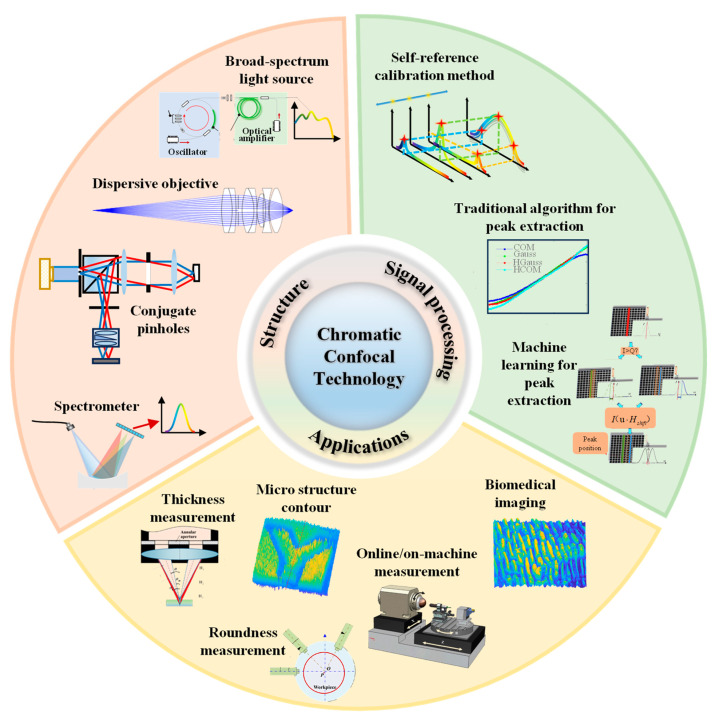
The main research interests of chromatic confocal technology.

This paper presents an overview of high-precision chromatic confocal sensing technology, as Figure 1 shows. It begins by introducing the main design principles and the structural components, such as the broad-spectrum light source, dispersion objectives, conjugate pinholes, and spectrometer. Then, the signal processing algorithms are described with the use of the self-reference calibration method, along with the traditional algorithm and the machine learning algorithm that are used for peak extraction. Furthermore, this paper explores various applications of chromatic confocal sensing technology, including thickness measurement, microstructure contour measurement, biomedical imaging, online or on-machine measurement, etc. Finally, this paper presents several challenges with measurement and a few potential directions to be explored and researched in the rapidly developing manufacturing industry.

## 2. Chromatic Confocal Technology

### 2.1. Principles

**Figure 2:**
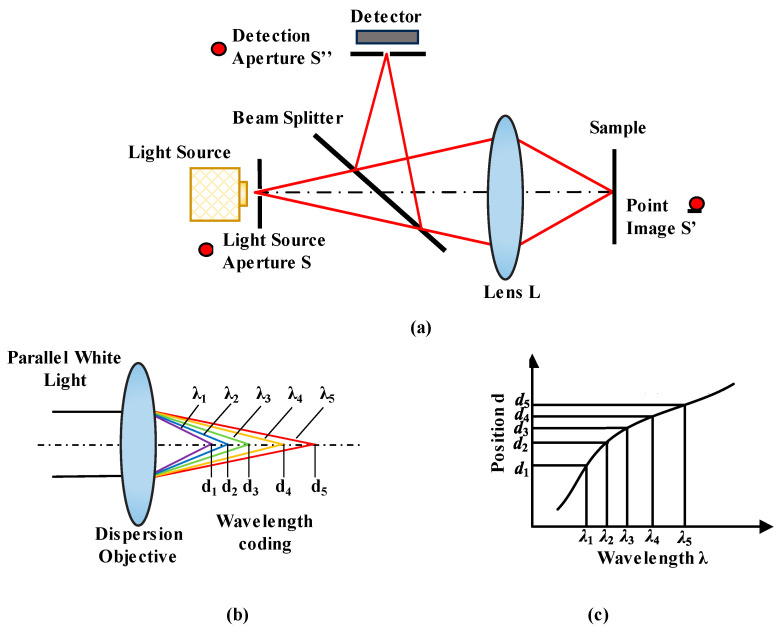
Principle of chromatic confocal technology. (**a**) Confocal optical path. (**b**) Wavelength coding. (**c**) Wavelength–position curve.

Chromatic confocal technology is similar to confocal microscopy. Confocal microscopy dates back to the 1940s and was developed by researchers such as Marvin Minsky [87]. To date, it has developed rapidly, giving birth to some significant branches, such as laser confocal microscopy and chromatic confocal technology. Laser confocal microscopy uses a monochromatic light source to form a single focus and thus achieve maximum light intensity during the axial scan, corresponding with the focus position. In contrast, chromatic confocal technology uses a broad light spectrum to form a series of foci, corresponding with different wavelengths. The imaging principle of chromatic confocal systems is illustrated in Figure 2. The broadband light source provides light through the first pinhole to allow the lens to focus on the sample, and the reflected light enters the detector through the detection aperture. Based on the optical principle, the light source aperture, the point image and the detection aperture conjugate with each other, as shown in Figure 2a. According to the dispersion phenomenon, different wavelengths of light will be focused on different axial positions by the ordinary lens because of different refractive indexes. Figure 2b shows the one-to-one relationship between wavelength *λ* and position *d*. By analyzing the wavelength of the light reflected from the sample surface, its position can be determined from the wavelength–position response curve shown in Figure 2c, which is calibrated before the measurement. 

Since the broadband spectrum of the light source can be divided indefinitely, it is feasible to organize the axial positions as densely as possible so as to realize high-resolution displacement measurements. Despite the accuracy limitations relating to the nonlinear response curve of optical components, the instability of light sources, and the extraction of the focus wavelength, the accuracy and stability of the chromatic confocal technology can usually be determined at the sub-micron level. However, its simple optical configuration helps to enhance the efficiency, integration, and applicability of the mirror, with rough or declining surfaces. Consequently, this has become one of the international standard methods used for displacement and profile measurements.

### 2.2. Broad Spectrum Light Source

Chromatic confocal technology uses a broad-spectrum light source for the wavelength encoding of the axial position. As a result, the continuity and the stability of the light source directly affect the resolution and stability of the displacement measurement. 

**Figure 3:**
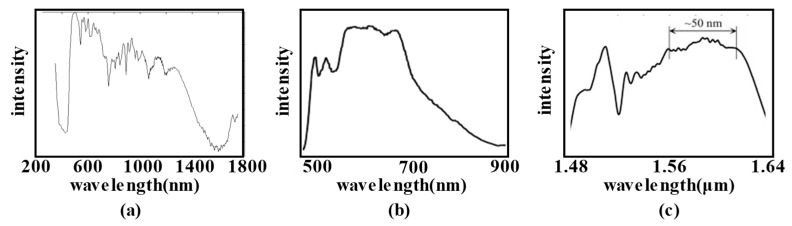
(**a**) The light source spectrum of PCF, adapted from [92]. (**b**) The spectrum of an MOF super-continuum light source, adapted with permission from [93], copyright 2013 Elsevier. (**c**) The spectrum of mode-locked femtosecond lasers, adapted with permission from [96], copyright 2018 Elsevier.

Early chromatic confocal technology used ordinary white light sources, such as tungsten halogen lamps, xenon lamps, white light emitting diodes (LEDs), etc. The white light intensity of the tungsten halogen or xenon lamp varies greatly in the visible band, resulting in large difficulties in signal processing over the full wavelength range. Further, their warm-up time is usually long, and their lifetime is short, meaning they are not suitable for most industry situations. White LEDs are widely used in chromatic confocal sensors, with the advantages of an appropriate spectral range (380~760 nm) and high light efficiency [88,89,90]. In addition, the super-continuum light source can also provide a broad spectrum based on nonlinear modulation. It has become a popular light source for use in chromatic confocal technology research because of its high brightness and high stability, and it is of great importance when seeking to reduce noise interference [91]. In Figure 3a, we see how K Shi et al. [92] obtained a super-continuum spectral light source (350~1750 nm) using a photonic crystal fiber (PCF) to enhance the efficiency and signal-to-noise ratio of the chromatic confocal sensor. In Figure 3b, it can be observed that U Minoni et al. [93] used a super-continuum spectral light source (488~1064 nm) produced by a micro-structured fiber (MOF) for the displacement measurement, with a 0.36% repeatability. H Liu and H Matsukuma et al. [94,95] utilized a similar super-continuum spectral light source (400~2400 nm) for their chromatic confocal imaging research. Beyond this, other researchers [96,97] have used mode-locked femtosecond lasers to achieve a high wavelength resolution between 1.46 and approximately 1.64 μm, as shown in Figure 3c.

In fact, commercial broadband light sources are now well-developed, with excitation by high-power blue lasers providing high-brightness continuous spectra to enable a wider measuring range. All of these broadband light sources provide equalized light intensity distribution and measuring stability, but the high power of the lasers, the complex structures, the high costs, and the specific safety risks make it difficult to apply them in batched industry fields. Meanwhile, simple, reliable, and low-cost LED white light sources are attracting increasing interest for both research on and commercial applications of chromatic confocal technology.

### 2.3. Conjugate Pinholes and Beam Splitter

**Figure 4:**
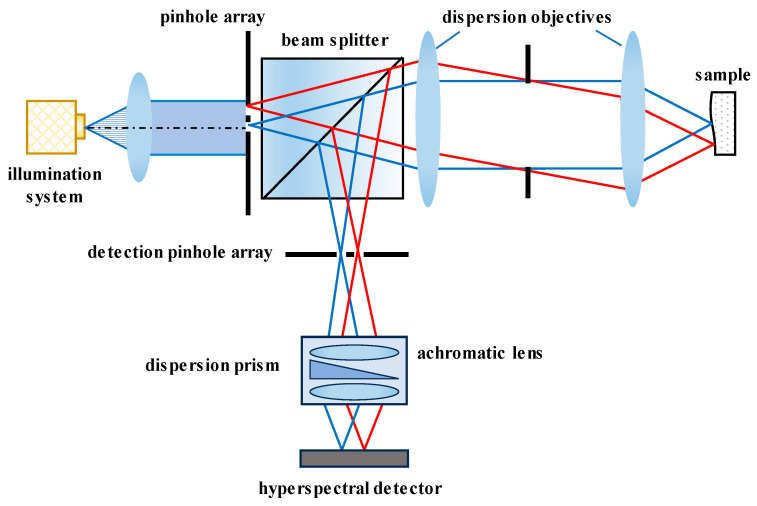
Chromatic confocal system with arrayed pinholes.

Conjugate pinholes are very important to the confocal phenomenon, and their size is crucial to controlling both the system resolution and the signal-to-noise ratio. Some researchers have analyzed the relationship between the aperture size and the resolution, detecting efficiency, etc. [98]. To increase the sample points in a single measurement, devices with multiple pinholes have been applied in chromatic confocal technology. For instance, Kar Tien [99] utilized a Nipkow disk in a confocal microscope between the beam splitter and dispersion objective to serve as both the light source aperture and the detection aperture for rapid contour scanning. Hwang J et al. [100] obtained high-contrast three-dimensional images by modulating the light frequency via a rotating disk aperture. On the other hand, arrayed pinhole structures demonstrate advantages in simultaneous multi-point contour measurements, as shown in Figure 4 [101,102,103]. The arrayed pinholes are also used together with a micro-lens arrays to diffract and focus light, thus improving the detection efficiency [104,105]. Qi Cui et al. [106] replaced arrayed apertures with two liquid crystal display matrices to realize computer control and enhance measuring flexibility. 

**Figure 5:**
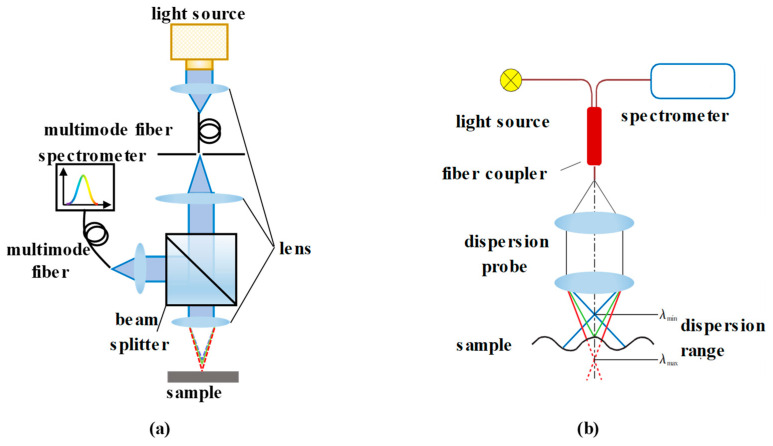
Fibers in chromatic confocal systems. (**a**) Multimode fiber and (**b**) fiber coupler.

Beyond the abovementioned pinhole structures, optical fibers are also a perfect choice to act as the pinhole because of their small core diameters [96,107,108,109]. These fibers can enable size customization, low signal loss, and stable beam transmission, as shown in academic research and commercial products. As Figure 5 illustrates, the optical fibers simplify the optical path, enable modular design and allow for the easy adjustment of the measuring parameters. In Figure 5a, a single multimode fiber is used to transmit the light while acting as a pinhole. In the structure shown in Figure 5b, the fiber coupler plays the roles of a beam splitter and the pinhole.

### 2.4. Dispersion Probe

In the method employing chromatic confocal technology, the dispersion probe is used to generate dispersion in order to code its axis with different wavelengths. This greatly affects the measuring range, permissible tilt angle, spot size, and even the response curve. In common, the wavelength–position response curve is obtained by calibration before actual measurement. A nonlinear response curve is mainly produced by optical components, requiring more calibration points to derive accurate results. Beyond this, the axial position resolution varies depending on the measuring range employed in deriving the nonlinear response curve, while it remains almost unchangeable when deriving the linear response curve. In common, a dispersion probe can be produced by use of a refractive lens, diffractive optical elements or metalens.

**Figure 6:**
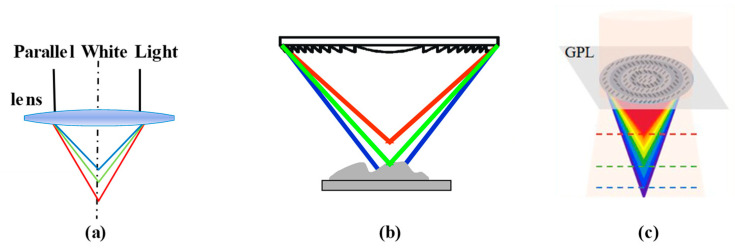
(**a**) Dispersion characteristics of refractive optical elements. (**b**) Dispersion characteristics of DOE, reprinted from [117]. (**c**) Dispersion characteristics of GPL, reprinted from [124].

Refractive lenses are mostly used in modern studies [110], employing the differences in refractive index of different wavelengths as they propagate through the transparent medium. In some early studies, white light was modulated to parallel light at first and then passed through the ordinary refractive lenses to achieve dispersion, using plano-convex or thin convex lens combinations, as shown in Figure 6a. However, this dispersion probe introduces extra dispersion in the collimation step, and it is difficult to optimize other aberrations aside from the chromatic aberration, leading to poor light spot quality on the target surface. Haodong Bai, Chunyan Li [111], Ailin Zhang [112], and Yanlei Li [113] designed integral dispersion probes with multiple lenses. Tingting Huang proposed an efficient optical design method for a line-scanning chromatic confocal displacement sensor [114].

In order to expand the dispersion range, researchers adopted a multi-lens group to achieve a maximum dispersion range of up to 30 mm [115]. However, this method increased the complexity of the dispersion probe and decreased the signal-to-noise ratio caused by the curving lens surfaces. To enhance the imaging quality of dispersion probes, Bai J used a spatial bandpass filter to reduce the influence of diffraction and decrease the focus spot’s size, leading to higher lateral resolution [116]. Furthermore, due to the high cost of customizing lenses, some researchers choose commercial dispersion lenses, whose lenses are usually composed of multiple layers and do not come with detailed parameters.

In addition, diffractive optical elements (DOEs) have begun to be used to generate dispersion. In Figure 6b, different wavelengths of light pass through the DOE at different angles for the same diffraction order in order to generate dispersion based on the diffraction principle. In contrast to refractive lenses, the focal spot produced by longer wavelengths is closer to that produced by the DOE [117]. A Fresnel lens is used as the dispersion lens in the studies of SL, J. Garzón [118], Rayer, M [119], and T Liu [120]. These studies explore the impacts of diffraction efficiency on wavelength–position response curves and the measuring resolution. Matthias Hillenbrand [121,122] compared the imaging effects of dispersion lenses composed of DOEs in a DOE lens group. DOEs have a simple structure, higher linearity in their wavelength–position response curves, and fewer optical surfaces. David [123], Aiko K, and P. Lücke et al. utilized DOEs to design and fabricate millimeter-sized dispersion elements for use in measuring microbore diameter. Moreover, DOEs have many adjustable parameters that provide flexible design. In addition, the metalens is a novel planar element used in the dispersion probe. In the structure shown in Figure 6c, a geometrical phase lens (GPL) is used to modulate the right-hand circularly polarized beam to focus on the axis [124]. Metalens-based optical designs have the potential to provide miniature, lightweight, low-cost optical sensors in future manufacturing [125]. 

In fact, it is well known that the depth of focus produced by diffraction depends on the focus wavelength and the numerical aperture of the optical elements. As such, the axial resolution cannot be decreased limitlessly. However, the light intensity along the focus depth varies in a fashion similar to a Gaussian curve, so it is easy to extract the ideal focus position using the centroid method, the parabolic fitting method, etc. Hence, actual axial positions can be effectively distinguished in spite of the focus depth.

### 2.5. Spectrum Detection

Because the axial position is extracted from the focus wavelength of the reflected light when using chromatic confocal technology, spectrum detection and peak extracting processes are essential. Generally, spectrum detection uses a prism or grating to decompose the chromatic light into monochromatic light, followed by the use of a photodetector to capture the intensity at various wavelengths, referred to as a spectrometer [126,127,128]. Commercial grating spectrometers are now well-developed for use in spectrum acquisition and analysis. They are usually integrated as tiny devices placed behind the detection fiber to reduce the size and weight of a chromatic confocal sensor. Before now, many kinds of miniature spectrometers have been introduced, with different manufacturing methods [129,130]. Interference lithography is one popular method used for fabricating gratings by use of light interference to generate periodic structures on a photoresist [131,132,133,134,135]. The grating quality usually determines the detection accuracy and spectral range of the spectrometer, such as the grating base and the period [136,137]. Concave mirror bases have been widely studied for use in miniature spectrometers [138,139,140]. The gratings period is determined by the beam angles, which depend on the spatial arrangement of the optical components [141]. Furthermore, the valid spectrum range can be broadened by employing a dual-slit configuration and mirrors at the grating edges in order to reuse the grating area [142,143]. Fresnel grating [144,145] is also a good choice for use in miniature spectrometers [146], one side of which is used for focusing light and the other side for dispersion. To improve its imaging quality, researchers designed a grating surface with variable pitch in order to improve its performance [147,148]. On the other hand, multi-channel spectrometers are attracting interest in the context of multi-point chromatic confocal measurement. Shan Shuonan [149] designed a microlens array grating to enable multi-point spectral detection in a chromatic confocal system, the measurement repeatability of which was only 0.8 μm [150].

**Figure 7:**
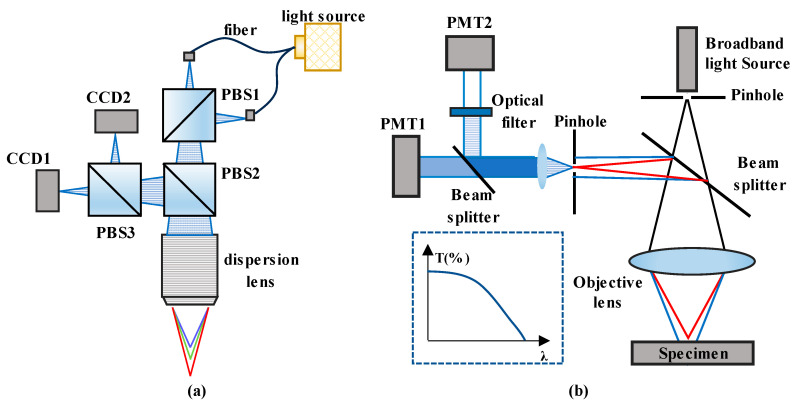
(**a**) Chromatic confocal system based on CCDs. (**b**) Reflected light detection based on transmittance.

Furthermore, some studies have explored the utilization of CCDs or color cameras for reflected light analysis. LiangChia Chen [151] used a polarization beam splitter (PBS) to form p-wave and s-wave beams to generate differential signals on two color CCDs (as shown in Figure 7a), after which the position could be derived from these signals by use of the proposed algorithm. Taejoong Kim [152] used transmittance to obtain the wavelength distribution, as shown in Figure 7b. Photomultiplier tubes (PMTs) require little time for integration when used to realize a high measuring speed. However, these detection methods can only identify certain spectral bands, and their wavelength resolution is relatively low, impacting the displacement-measuring resolution in chromatic confocal systems.

## 3. Spectrum Analyzing

### 3.1. Normalization and Peak Extraction

Chromatic confocal sensors obtain the sample position by analyzing the reflected spectrum, which is usually mountain-shaped, whereby the peak represents the focus wavelength with the biggest intensity. G. Molesini [153] and Kebin Shi et al. [154] studied the peak extraction algorithm, but did not take into account the influence of the broad spectrum. In practice, although the confocal pinhole blocks the majority of non-focused light, some extra reflected light may still reach the detector. Thus, the reflected light spectrum easily broadens and deviates from its ideal peak. As the pinhole size increases to increase the amount of reflected light, it becomes more difficult to extract the peak wavelength. 

Therefore, spectra normalization is usually used to alter the reflected spectrum into its ideal mountain shape in order to improve peak extraction accuracy. In fact, the intensity distribution of the reflected spectrum mostly depends on the light source and the sample surface [155]. Spectrum normalization methods have been developed that use the light source spectrum or the reflected light spectrum as the reference spectrum. Shuai Wang [156] proposed the use of a virtual double-slit differential chromatic LCI to decrease the FWHM and achieved a higher axial resolution. Nouira H [157] and Yu Q [158] analyzed the influences of the sample material, color, roughness, etc. They compensated for the presence of different colors in the reflected spectrum of the tested surface in order to decrease the measuring errors. Mengmeng Xi [159,160] analyzed the peak wavelength shift of the reflected spectrum, and compensated for it with calibration. J Bai [161] proposed a self-reference method to pre-scan the sample surface and then correct the reflective differences of the tested surfaces. 

After spectrum normalization, peak wavelength extraction is performed to obtain the focal wavelength from the reflected light spectrum. If the full width at half maximum (FWHM) is narrow, the maximum intensity and centroid methods are preferred [154,162,163]. Although these methods are advantageous for use in online measurements, due to their simplicity and efficiency, their extraction accuracy is limited as a result of out-of-order spectrum fluctuation. The curve fitting method is another peak extraction method with higher accuracy, involving parabolic fitting, sinc2 curve fitting, etc. Niu Chun Hui [164] and Ding Luo [107] compared these peak extraction algorithms, including the centroid method, the Gaussian fitting method [165], the threshold centroid method, and the threshold Gaussian fitting method. The extraction accuracy of the Gaussian fitting method was found to be the highest, and the modified difference fitting and the mean shift methods showed improved efficiency [108,166]. Moreover, recent studies have employed machine learning algorithms, such as the general regression neural network (GRNN), to precisely derive the peak wavelength [167]. In addition, Jiacheng Dai [168] compensated for the nonlinear error in the response curve with a regression-tree model to improve the measurement accuracy by 40%.

### 3.2. Signal Processing of Dual-Detection Chromatic Confocal Probe

**Figure 8:**
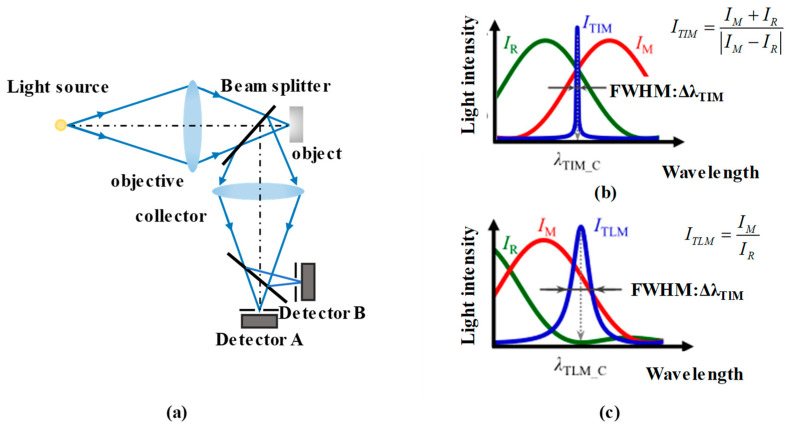
(**a**) The schematic of a dual-detection configuration. (**b**,**c**) The methods used to extract the peak value, with differential type and ratio type.

In chromatic confocal technology, the wavelength–position response curve depends on the probe’s dispersion performance. There is a tradeoff between the measurement’s resolution and its range [169]. Therefore, a dual-detection configuration is proposed to achieve a high-contrast signal, as shown in Figure 8a. The light reflected from the sample’s surface is divided into two beams for the two detectors. The two detected signals, I_A_ and I_B_, can be used to calculate the ratio I_R_ = I_A_/I_B_ and the difference I_M_ = I_A_ − I_B_. Figure 8b,c show the two kinds of signal processing algorithms that can be applied to the target spectra to extract the confocal wavelengths [170]. Clearly, the algorithm in Figure 8b provides a smaller FWHM than that in Figure 8c. 

## 4. Applications

### 4.1. Contour Measurement

**Figure 9:**
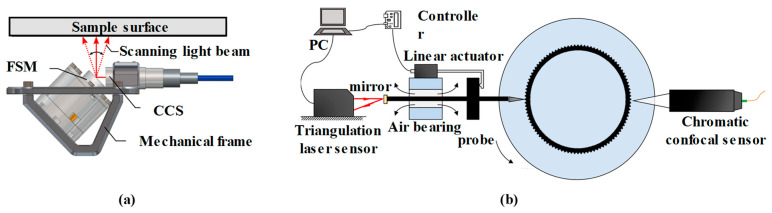
(**a**) The chromatic confocal sensor combined with fast steering mirror (FSM), reprinted from [175]. (**b**) The measurement system using a chromatic confocal sensor to obtain the profile of the micro gear teeth, reprinted from [176].

A chromatic confocal sensor can rapidly measure submicron-level displacements. Furthermore, it can measure the relative heights of multiple points along the scan path. By subtracting the reference plane, the surface profile of the tested sample can be obtained. This approach has diverse applications in industrial fields. Fu Shaowei [171] used a chromatic confocal sensor for roughness detection. V. Rishikesan Lishchenko N [172] achieved offline roughness measurements of turned and milled surfaces with a chromatic confocal sensor. The scanning results match well with those produced by a profilometer and show a superior lateral resolution and ability to capture profile details. Nadim El Hayek [173,174] utilized chromatic confocal sensors to measure transparent curving sample surfaces. In the aforementioned research, the sample or probe was fixed onto a displacement stage for the scan. In some studies, additional devices, such as fast steering mirrors (FSMs), were used for scanning, as Figure 9a shows [175]. Huang GY [176] proposed a hybrid strategy to overcome the challenge of measuring micro-gear teeth with a small modulus, as shown in Figure 9b. The contact probe captured the falling flank profiles in segments, using an auxiliary lifting mechanism to avoid interference on the rising slope. The non-contact chromatic confocal displacement sensor accurately measured the gear peak positions via the two-point error separation method. With the flank profile data and gear peak positions, a full gear profile was obtained. Yifu Wan [177] developed a four-axis measurement system with a chromatic confocal sensor to successfully measure a spherical specimen with a radius of 50 mm.

**Figure 10:**
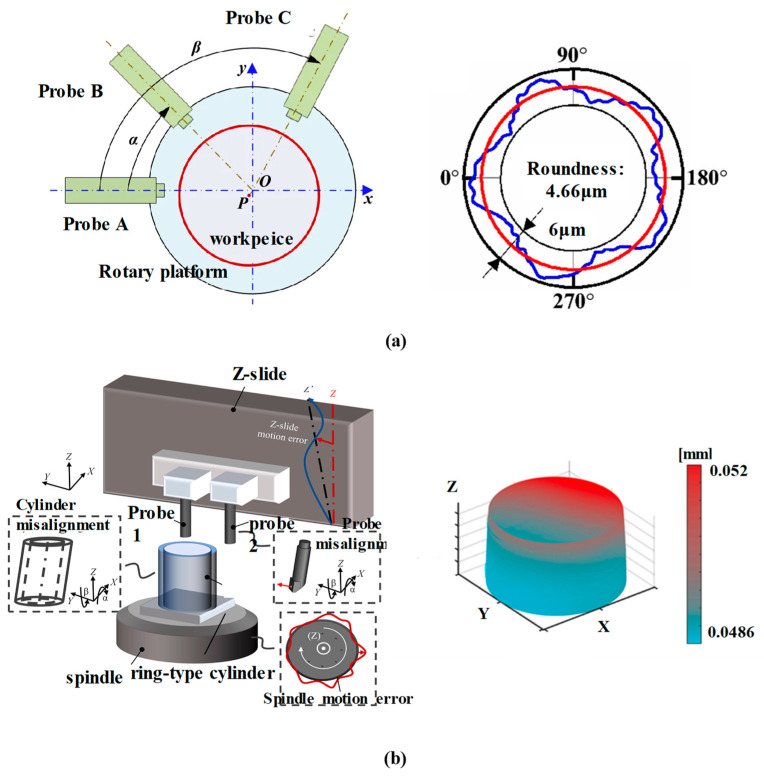
Roundness measurement. (**a**) Three-probe system and its measuring result, reprinted from [180]. (**b**) Dual-probe system and its measuring result, reprinted with permission from [181], copyright 2022 Elsevier.

Roundness can also be measured using multiple chromatic confocal probes. With the optimization of measurement strategies, the precision can reach sub-micron levels, making it comparable to other high-precision instruments. Qu Dingjun [178] applied a line-scanning chromatic confocal sensor to measure wafer eccentricity deviation. This eccentricity deviation measurement accuracy was insensitive to noise and reached the micron level. Esmaeil Heidari [179] used chromatic confocal probes to measure the semi-cylindrical thin plate workpiece, and obtained profiles and surface roughness values for both convex and concave surfaces. The measurement process was flexible and simple, and the results match the standard values. J Bai [180] used three chromatic confocal probes to obtain the outer profiles and the roundness of cylindrical components via the three-point method, as shown in Figure 10a. After error separation, the measurement results were found to be consistent with those produced by the ultraprecise roundness instrument. Xin Xiong [181] designed a two-probe chromatic confocal system, as shown in Figure 10b, to minimize the positioning and tilt errors in geometric form measurements of cylindrical parts with a rotating spindle. The results show good correspondence with those produced by the coordinate measuring machine (CMM). The measurement uncertainties were on the sub-micrometric level. Li QL [182] acquired two-dimensional coordinates of a superfine cylinder surface with a chromatic confocal sensor.

**Figure 11:**
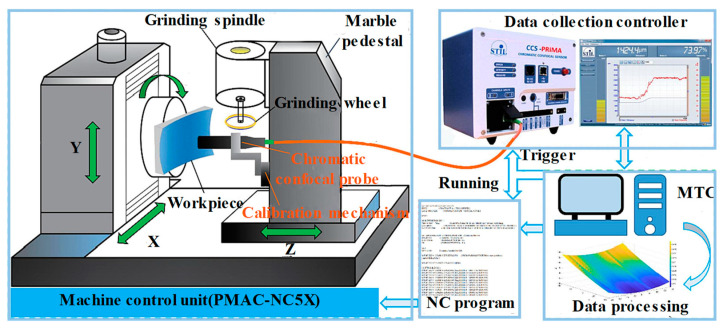
On-machine measurement, reprinted with permission from [181], copyright 2022 Elsevier.

**Figure 12:**
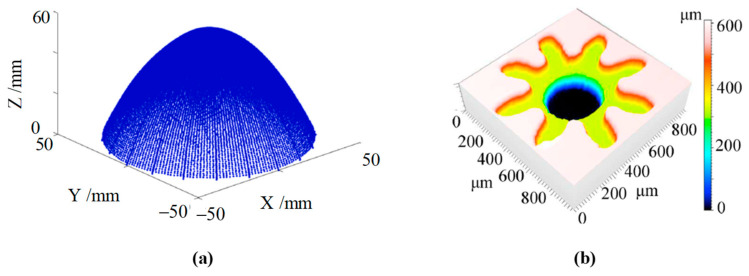
The results of on-machine contour measurement. (**a**) Steep surface, reprinted with permission from [185], copyright 2022 Elsevier. (**b**) Micro-gear, reprinted with permission from [189], copyright 2021 Elsevier.

Chromatic confocal systems are also widely used for in-process or online surface profile measurements. In ultra-precision machining, processing and inspecting free-form surfaces pose significant challenges. The advantage of using a chromatic confocal sensor for slope surface measurement is that it can be integrated into the ultra-precise machine for detection and error compensation before or after manufacturing [183,184,185], as shown in Figure 11. The manufactured workpiece was successfully measured, with its slope surface shown in Figure 12a. X Zou [186] obtained three-dimensional profiles of machined convex surfaces and periodic micro-structures, with an uncertainty of only 83 nm. Sheng Wang [187] measured the profiles of off-axis free-form optics on an on-machine system to improve the machine efficiency and reduce the production cost. Ye Long [188,189] investigated the uncertainty of an on-machine measuring device with a chromatic confocal sensor. They revealed the most influential factors, such as the vibration disturbances, and provided a systematic quantification of uncertainties. In Figure 12b, we show a micro-gear contour that was constructed with an area of 1 mm × 1 mm. Bin Zhao [190] integrated a chromatic confocal sensor with a pressure sensor, and achieved real-time micrometer-level measurement of the deformations inside the sealed cavity.

Although lateral scanning can help to realize contour measurements, it is usually limited by the moving stages and the data processing ability. Hence, the conjugate pinhole array, DMD, or microlens are used in the chromatic confocal system to form a point light array on the surface for multi-point measurement. This configuration significantly enhances the measuring efficiency. KT Ang [99] and Prause, K [191] conducted some research to obtain three-dimensional profiles of micro-columns and micro-grooves. Additionally, some researchers [175,192,193,194] have utilized rotated mirrors to scan the profile and thus improve the measurement efficiency. However, the horizontal resolution is usually limited by the spacing between the pinholes. Additionally, array-shaped elements make the optical path susceptible to diffuse reflections produced by surface irregularities, and the measurement area is usually smaller than that offered by the scanning method.

### 4.2. Biomedical Imaging

**Figure 13:**
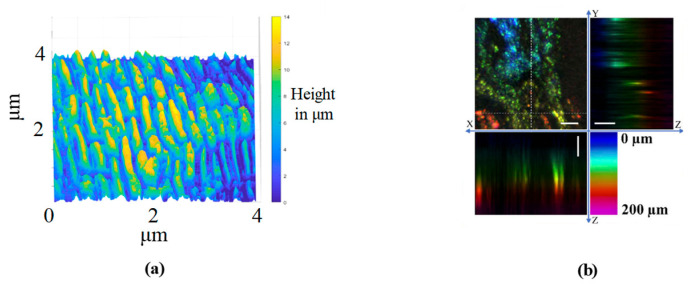
Offline detection of biological tissues using spectral confocal technology. (**a**) Onion slice, reprinted from [106]. (**b**) Ex vivo cancerous tissue, reprinted from [198].

The use of chromatic confocal technology is a non-destructive measurement technique that can be applied to contour measurements in biomedicine, without applying heat to or destroying the biological tissues. Yang, X F [195] used a chromatic confocal probe to scan multi-depth volumetric nude-mouse skin. As shown in Figure 13a, Qi Cui [106] used a chromatic confocal sensor to achieve the three-dimensional imaging of onion slices with a height resolution of 1.3 μm. M. Zint [196] utilized a chromatic confocal system with a microlens array and a pinhole array to obtain high resolution and accuracy in dental contour measurements at a reduced cost. Johnson Garzón R Kübler [197] has employed a chromatic confocal sensor in lateral scanning to measure the central thickness of the human cornea and retina. Yang, X [198] performed imaging of human gastric tissues. Cory Olsovsky [199,200] employed a non-spherical lens to achieve a dispersion range of 150 μm for the rapid three-dimensional imaging of porcine oral mucosa before cell apoptosis.

### 4.3. Thickness Measurement

**Figure 14:**
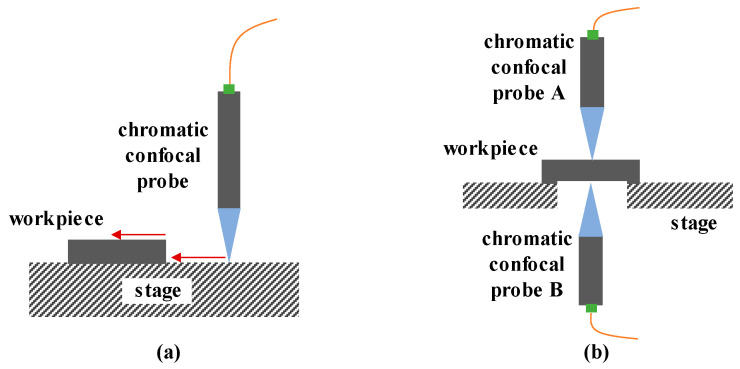
The methods used to measure the thickness of an opaque workpiece. (**a**) Single probe and (**b**) dual probes.

There are two common methods used to measure the thickness of thin, solid workpieces. As shown in Figure 14a, the workpiece is placed on a flat surface for its step height measurement. Figure 14b shows two symmetrical probes set above and below the workpiece to determine the thickness from the distance between the two axes.

**Figure 15:**
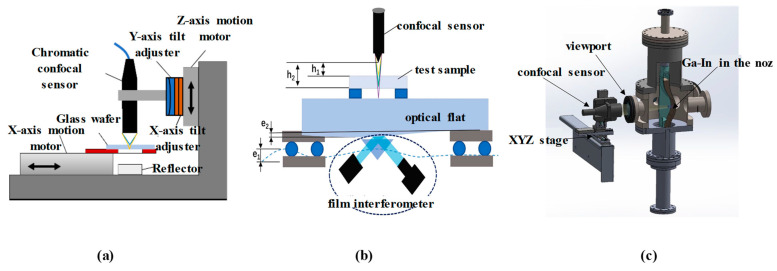
(**a**) A method for measuring the thickness of transparent workpieces, reprinted from [181]. (**b**) Thickness measurement using a chromatic confocal sensor and interferometer, reprinted with permission from [184], copyright 2023 Elsevier. (**c**) The liquid thickness measurement, reprinted with permission from [187], copyright 2023 Elsevier.

On the other hand, the film thickness of transparent samples can also be measured by use of chromatic confocal technology. The reflected light forms two peaks in the reflection spectrum, relating to the upper and lower film interfaces [201]. Hence, the axial position interval can be derived from two peaks to determine the film thickness [202]. Under this method, the refractive indexes of transparent samples influence the film thickness measurement results. In Figure 15a, an optimized model is proposed to measure the film thickness without the need to use the refractive index by employing a motor to drive the sensor and placing a reflector behind the sample. Chunmin Liu [165] designed a chromatic confocal sensor for online measurement to obtain the thickness of transparent samples. Niese and Svenja [203] also integrated chromatic confocal sensors into the production line to help in measuring the polymer film and tempered glass thickness. Fang Cheng [204] proposed a 3D surface scanning system as a platform for the inspection of a sapphire substrate. As shown in Figure 15b, the chromatic confocal probe here obtains the position with compensation for the error produced by the interferometer. Christian Haider [205] introduced a strategy to overcome the tradeoff between the thickness measurement range and the lateral resolution of a chromatic confocal sensor. Jiafu Li [206] proposed a novel measurement model by adding an auxiliary transparent film into the dispersion field to avoid the use of a standard workpiece. Additionally, the thickness of the liquid film can be measured using chromatic confocal sensors. I. Eliyahu [207] measured the thickness of a gallium indium free surface jet, and obtained high accuracy and resolution using low-cost, non-contact chromatic confocal sensors, as shown in Figure 15c. Jiantao Lan [208] employed a chromatic confocal system to observe the evaporation dynamics of sessile water droplets under various environmental conditions, showing its potential use in experimental fluid dynamics. Beyond this, J Bai used a chromatic confocal sensor to measure the spectral reflectance of a film and fit it with the theoretical reflectance to determine its thickness [209]. The measurement accuracy of this approach is comparable to that of a reflectometer, and the lateral resolution is higher, enabling micro-area film thickness measurement. Similarly, Ching-Te Kuo [210] et al. measured a pellicle surface by analyzing the reflected light wavelength, with an axial resolution of 22 nm.

## 5. Conclusions

High-precision displacement measurement plays a key role in the modern advanced manufacturing industry. In summary, chromatic confocal technology is now rapidly developing, with numerous applications in both industrial areas and scientific research. The chromatic confocal sensor consists of a broad-spectrum light source, a dispersion probe, conjugate pinholes, a spectral detection unit, and signal-processing devices. All of these components have attracted interest for development, including super-continuum light sources, diffractive lenses, pinhole arrays, etc. In signal processing, machine learning methods have been introduced to enhance measurement performance. The introduction of the dual-probe structure has further broadened the measurement range of chromatic confocal sensors. On the other hand, studies employing displacement measurement, contour measurement, thickness measurement, etc. have been proven to be especially advantageous for application to tilted surfaces, rough surfaces, mirrors, and even curved surfaces. The high speed and stability of these methods make them suitable for integration into precision machining equipment used for online, on-machine processing measurements.

Lastly, some challenges remain to be solved. The design methodology used for chromatic confocal sensors should be more flexible in order to take into account the dispersion range, spot size, response curve linearity, and detection efficiency. The diffractive optical element shows a linear response curve but decreased light efficiency. The light source spectrum’s distribution is always rugged, impacting the signal-to-noise ratio at out-of-focus wavelengths, thus producing systematic errors in focus wavelength extraction. Further, spectrum normalization also needs to be further explored so as to correct for light fluctuation and sample surface variance. It is urgently required to enhance the measurement efficiency of multi-point chromatic confocal measurements, which may require the use of multi-channel spectroscopic detection devices. The design and fabrication of the gratings in these devices are still challenging. Additionally, signal processing using machine learning algorithms may be a good choice in order to enhance the performance of the chromatic confocal sensing technology used. Finally, it is suggested that the applications of chromatic confocal sensors be expanded to some new fields, such as color detection, thin film thickness measurement, etc.
